# Supplementary material for: The Combined Immunohistochemical Expression of GLI1 and BCOR in Synovial Sarcomas for the Identification of Three Risk Groups and Their Prognostic Outcomes: A Study of 52 Patients
Source: Int J Mol Sci. 2024 Jul 11;25(14):7615. doi: 10.3390/ijms25147615 (PMC11276717; doi:10.3390/ijms25147615)
Supplement: Supplementary file 1 [file ijms-25-07615-s001.zip › Table S1.pptx]

## Slide 1
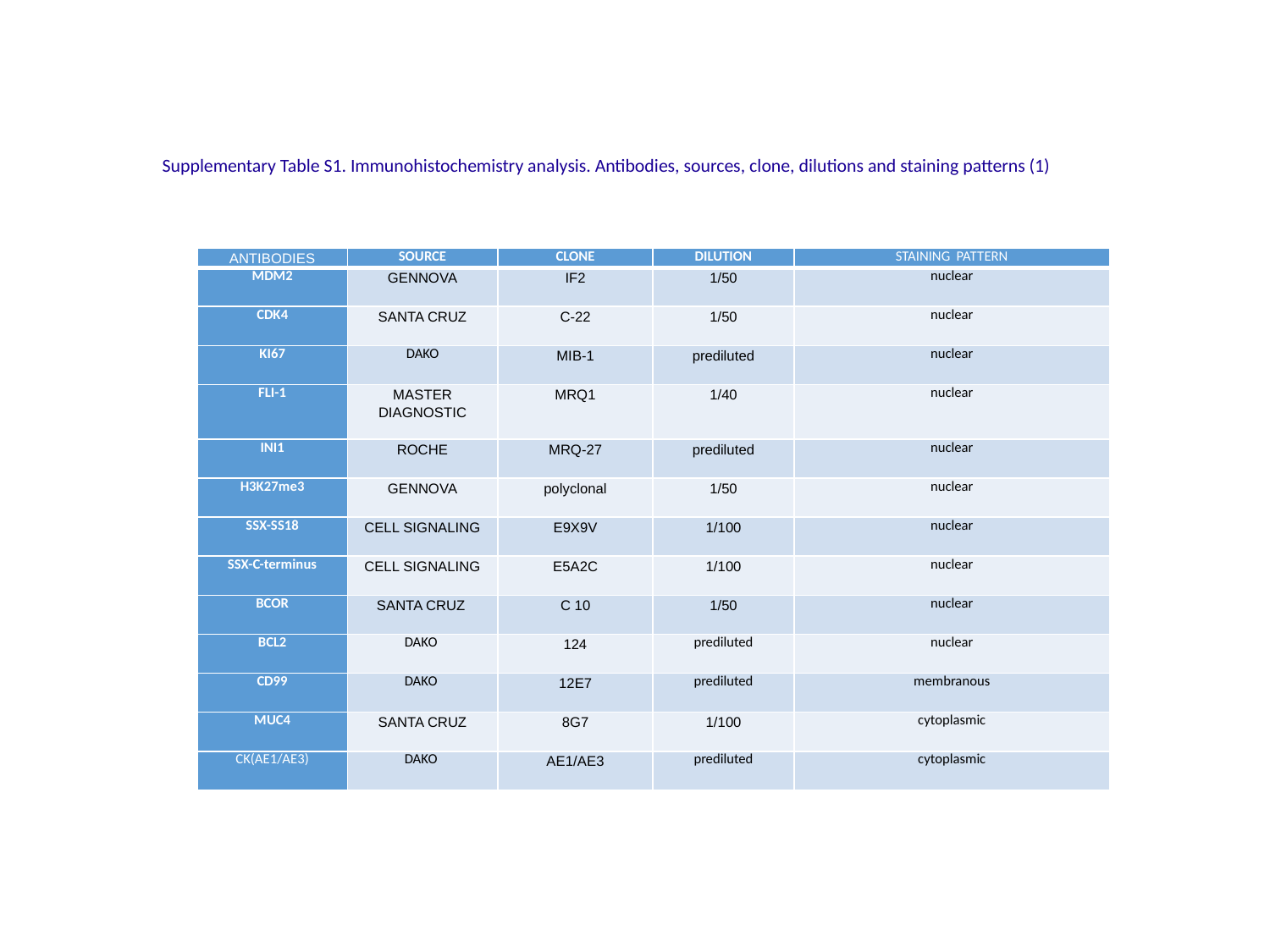

Supplementary Table S1. Immunohistochemistry analysis. Antibodies, sources, clone, dilutions and staining patterns (1)
| ANTIBODIES | SOURCE | CLONE | DILUTION | STAINING PATTERN |
| --- | --- | --- | --- | --- |
| MDM2 | GENNOVA | IF2 | 1/50 | nuclear |
| CDK4 | SANTA CRUZ | C-22 | 1/50 | nuclear |
| KI67 | DAKO | MIB-1 | prediluted | nuclear |
| FLI-1 | MASTER DIAGNOSTIC | MRQ1 | 1/40 | nuclear |
| INI1 | ROCHE | MRQ-27 | prediluted | nuclear |
| H3K27me3 | GENNOVA | polyclonal | 1/50 | nuclear |
| SSX-SS18 | CELL SIGNALING | E9X9V | 1/100 | nuclear |
| SSX-C-terminus | CELL SIGNALING | E5A2C | 1/100 | nuclear |
| BCOR | SANTA CRUZ | C 10 | 1/50 | nuclear |
| BCL2 | DAKO | 124 | prediluted | nuclear |
| CD99 | DAKO | 12E7 | prediluted | membranous |
| MUC4 | SANTA CRUZ | 8G7 | 1/100 | cytoplasmic |
| CK(AE1/AE3) | DAKO | AE1/AE3 | prediluted | cytoplasmic |

## Slide 2
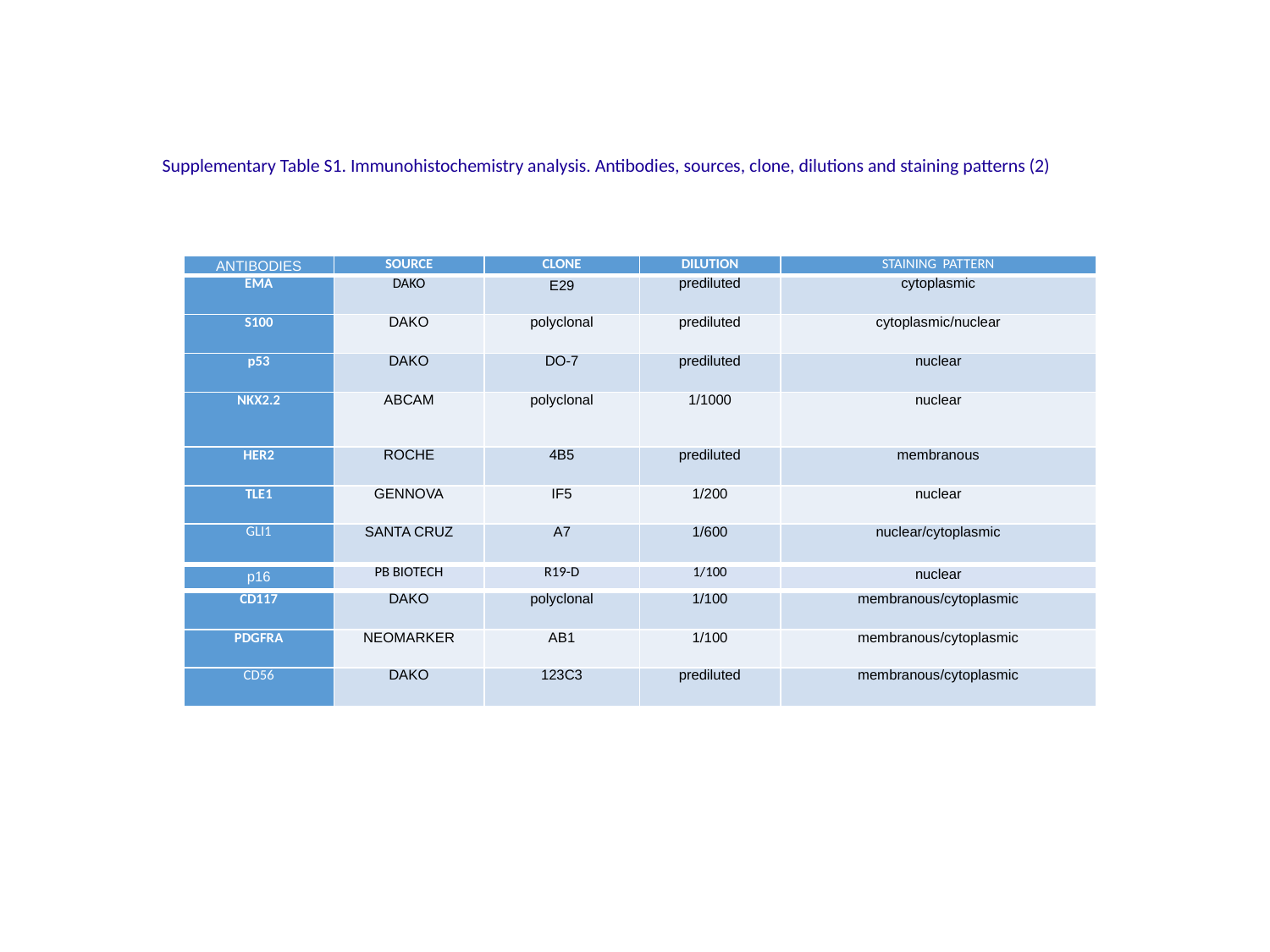

Supplementary Table S1. Immunohistochemistry analysis. Antibodies, sources, clone, dilutions and staining patterns (2)
| ANTIBODIES | SOURCE | CLONE | DILUTION | STAINING PATTERN |
| --- | --- | --- | --- | --- |
| EMA | DAKO | E29 | prediluted | cytoplasmic |
| S100 | DAKO | polyclonal | prediluted | cytoplasmic/nuclear |
| p53 | DAKO | DO-7 | prediluted | nuclear |
| NKX2.2 | ABCAM | polyclonal | 1/1000 | nuclear |
| HER2 | ROCHE | 4B5 | prediluted | membranous |
| TLE1 | GENNOVA | IF5 | 1/200 | nuclear |
| GLI1 | SANTA CRUZ | A7 | 1/600 | nuclear/cytoplasmic |
| p16 | PB BIOTECH | R19-D | 1/100 | nuclear |
| --- | --- | --- | --- | --- |
| CD117 | DAKO | polyclonal | 1/100 | membranous/cytoplasmic |
| PDGFRA | NEOMARKER | AB1 | 1/100 | membranous/cytoplasmic |
| CD56 | DAKO | 123C3 | prediluted | membranous/cytoplasmic |
